# DNR Linked to Reduced Depressive Symptoms in Nursing Home Residents During COVID-19 Illness

**DOI:** 10.1093/geroni/igab046.2668

**Published:** 2021-12-17

**Authors:** Wingyun Mak, Orah Burack, Joann Reinhardt, Himali Weerahandi, Benjamin Canter, Kenneth Boockvar

**Affiliations:** 1 The New Jewish Home, New York, New York, United States; 2 NYU Grossman School of Medicine, New York, New York, United States; 3 The New Jewish Home, The New Jewish Home, New York, United States

## Abstract

Prior work shows that older adults who establish future care plans have a lower risk of depression. Residents in long-term care may benefit from establishing a do-not-resuscitate (DNR) order when cardiopulmonary resuscitation is unlikely to provide medical benefit. The current study examines whether having a DNR order in place prior to COVID-19 diagnosis was associated with fewer depressive symptoms during the illness course. Residents at a NYC skilled nursing facility with a positive COVID-19 PCR test between 3/1/2020 – 6/1/2020 were included (N=338). The Minimum Data Set (3.0) was used to examine residents’ Patient Health Questionnaire-9 (PHQ-9) scores 1-30 days after diagnosis, functional status, cognition, age, and sex. A retrospective chart review was conducted to determine whether participants had an established DNR, DNI, and/or DNH order before developing COVID-19. Forty-eight percent, 46%, and 12% of participants had a DNR, DNI, or DNH order prior to COVID-19 illness, respectively. Average PHQ-9 score was 1.65 (SD=2.37). A hierarchical regression showed that after controlling for age (β=-.13, p=.06), sex (β=-.08, p=.28), cognition (β=.14, p=.04), and functional status (β=.23, p=.001; R2=.10, p=.001), having a DNR (β=-.22, p=.006) order in place prior to COVID illness was associated with lower endorsement of depressive symptoms during illness (ΔR2=.04, p=.01). Results suggest that establishing a DNR in long-term care residents when appropriate may potentially buffer depressive symptoms during illness in nursing home residents regardless of their age, sex, cognitive abilities, and functional status. Future examination of the underlying mechanism is warranted.

